# Associations of cardiorespiratory fitness, body composition, and blood pressure with arterial stiffness in adolescent, young adult, and middle-aged women

**DOI:** 10.1038/s41598-022-25795-x

**Published:** 2022-12-09

**Authors:** Eero A. Haapala, Earric Lee, Jari Karppinen, Hannamari Skog, Maarit Valtonen, Urho M. Kujala, Jari A. Laukkanen, Johanna K. Ihalainen, Eija K. Laakkonen

**Affiliations:** 1 Faculty of Sport and Health Sciences, University of Jyväskylä, Jyvaskyla, Finland; 2 Institute of Biomedicine, University of Eastern Finland, Kuopio, Finland; 3 Finnish Institute of High Performance Sport KIHU, Jyväskylä, Finland; 4 Institute of Clinical Medicine, School of Medicine, University of Eastern Finland, Kuopio, Finland; 5 Department of Medicine, Jyväskylä, Central Finland Health Care District, Finland District, Jyvaskyla, Finland; 6 Gerontology Research Center, University of Jyväskylä, Jyvaskyla, Finland

**Keywords:** Biomarkers, Cardiology, Medical research, Circulation

## Abstract

Few studies have investigated whether higher cardiorespiratory fitness (CRF) or favourable body composition are related to lower arterial stiffness in women. We therefore investigated the associations of CRF, body fat percentage (BF%), fat free mass index (FFMI), and mean arterial pressure (MAP) with arterial stiffness in 146 women aged 16–58 years. CRF was assessed by a maximal exercise test with respiratory gas analysis either on a cycle ergometer or a treadmill. Aortic pulse wave velocity (PWVao), augmentation index (AIx%), and MAP were assessed by a non-invasive oscillometric device and BF% and FFMI by a bioelectrical impedance or DXA device. CRF was inversely associated with PWVao (β =  − 0.004, 95% CI − 0.005 to − 0.002) and AIx% (β =  − 0.075, 95% CI − 0.102 to − 0.048) and these associations remained similar after adjustment for BF% or MAP, but not after the adjustment for age. FFMI was inversely associated with PWVao (β =  − 0.010, 95% CI − 0.019 to − 0.002) and MAP directly associated with PWVao (β = 0.005, 95% CI 0.003 to 0.006) and AIx% (β = 0.092, 95% CI 0.069 to 0.116) and the associations with PWVao also remained after further adjustments for BF% and age. In conclusion, a higher FFMI and a lower MAP were independently associated with lower arterial stiffness.

## Introduction

Arterial stiffness is an early sign of cardiovascular diseases that can be observed already in children and adolescents^1^. Increased arterial stiffness also independently increase the risk of incident cardiovascular events and cardiovascular mortality^2^. Arterial stiffness usually increases with ageing mainly due to fractured elastin and increased collagen content in the arterial wall^3^. Furthermore, with age, cardiorespiratory fitness (CRF)^4^ and fat free mass (FFM) decrease^5,6^ while fat mass (FM)^5,6^ and blood pressure^7^ increase. These changes, if accentuated, may also accelerate arterial stiffening during the life-course^8,9^. However, only a few studies have investigated whether decreased CRF and FFM and increased FM and blood pressure are associated with higher arterial stiffness in middle-aged compared to younger individuals.

Some evidence suggests that at least part of the age-related arterial stiffening in children^1,10^, adolescents^11,12^, and adults^13–16^ could be prevented by maintaining high CRF, but the evidence is inconclusive^17–19^. Continuous use of CRF scaled by whole body mass (BM) in previous studies may have clouded the true associations between CRF and arterial stiffness because CRF scaled by BM introduces a confounding by adiposity^20,21^. Accordingly, increased adiposity and accompanying hypertension have been associated with higher arterial stiffness in various age-groups^10,17^. Furthermore, a low amount of muscle mass has been associated with increased arterial stiffness in middle-aged and older adults^22–24^, but there are few studies in younger age-groups^25^.

Most previous studies have investigated the associations of CRF, body composition, and blood pressure with arterial stiffness in samples including both women and men^11–15^. However, women differ from men in terms of a unique natural maturation-dependent development of CRF^26^, body composition^27^, and hormonal concentrations^28^ affecting vascular health. During puberty, CRF does not increase in women while men continue to increase their CRF until young adulthood^26,29^. Women also accumulate more FM and less FFM than men leading to a higher body fat percentage (BF%) among women^27,30^. These differences in CRF and body composition remain during adulthood^31,32^. Nevertheless, women often demonstrate a lower arterial stiffness than age-matched men before the menopause^9,28^. These sex-differences have been thought to be partly attributed to the protective effects of oestrogen on women’s vasculature^28^. Therefore, more studies in representative populations among women are warranted to provide evidence on the associations of CRF, body composition, and blood pressure with arterial health.

The main aim of this study was to investigate the associations of CRF with arterial stiffness in a sample including adolescent, young adult, and middle-aged women. We also examined whether the associations of CRF with arterial stiffness are modified by BF%, mean arterial pressure (MAP), or age. Because CRF has been considered as a powerful marker of health in various populations independent of several other risk markers, we specifically studied whether middle-aged women have similar arterial stiffness and MAP than adolescents and young adults with comparable levels of CRF. Finally, we studied the associations of FFM, BF%, and MAP with arterial stiffness.

## Methods

### Design and participants

The present analyses are based on the data from four separate studies among women in their adolescence (The Neural Effects of Exercise, Diet, and Sleep (NEEDS) study^33^), young adulthood (The Endogenous and exogenous hormones and performance in women (MEndEx) study^34^, Monitoring Injury and Illness in Athletes (MIIA) Study, and middle adulthood (The Estrogen, MicroRNAs and the Risk of Metabolic Dysfunction (EsmiRs) study^35^.

In the NEEDS study, 55 apparently healthy 16–19-year-old adolescents (19 boys and 36 girls) were recruited from high schools and vocational schools located in the city of Jyväskylä, Finland^33^. We included only the female participants for the current analyses. In the MEndEx Study^34^, healthy women aged 18–40 years were recruited by advertisements in the local newspaper and via social media. Inclusion criteria required a participant to be physically highly active (training 6 times a week) with a body mass index (BMI) of 18–25 kg/m^2^. In the MIIA study, a total of 120 young female athletes aged 16–30 years were enrolled for prospective 2-year injury and illness monitoring that included body composition, energy availability assessment, and fitness testing. Inclusion criteria required a participant to be over 15 years old and compete in her sport. This study includes only the participants (n = 74) with arterial stiffness assessments. The participants in the EsmiRs study were recruited from the earlier ERMA Study^36^ comprised of a population sample of 47–58-years old women. EsmiRs performed a 4-year follow-up study for the ERMA study. This study uses only women (n = 37) who participated CRF and arterial stiffness measurements of the EsmiRs study^35^. One participant turned out to be voluntarily on a strict low energy and carbohydrate diet resulting in ketosis, hence she was excluded from the study. The valid data for the current analyses was available for 36 women.

In all studies, participants were excluded if they had conditions affecting the safety of exercise testing or regularly used medications that affect body metabolism, including medications for diabetes, thyroid dysfunction or dyslipidemia treatment. Regular use of sedatives, analgesics and alpha- or beta-blockers also led to an exclusion.

The protocols of the NEEDS (8/2016) and the MEndEx (10/2018) studies were approved by the ethics committee of the University of Jyväskylä, Finland. The Ethics committee of the Central Finland Health Care District approved the EsmiRs (9U/2018) and the MIIA (5U/2019) studies. All studies adhered to the Declaration of Helsinki except for registration to a publicly accessible database before recruitment. Participants gave their informed consent.

### Assessment of body size and composition

Stature was measured in the Frankfurt plane without shoes by a standard stadiometer. Body composition was measured by bioelectrical impedance analysis by InBody 720 device (Biospace Co. Ltd., Seoul, South Korea) using standard protocols^33^ and the participants wearing only their undergarments. The InBody device provides estimates of FM and FFM and BF% (FM (kg)/BM (kg) × 100). BM was assessed either by the InBody device or by the beam scale. In the MIIA Study, we used body composition data from the DXA (LUNAR Prodigy Advance with Encore software version 9.3, GE medical systems, USA) scans for 14 participants because they did not have InBody data available. Fat free mass index (FFMI) was computed as FM (kg)/stature (m)^2^ and as FFM (kg)/stature (m)^2^. While a reasonable agreement between these two methods have been reported previously^31,37^. InBody has been found to underestimate FM (mean difference (md) =  − 1.08 kg, limits of agreement (la) =  − 0.276 to 0.59 kg in girls and md =  − 3.1 kg, la =  − 6.7 to 1.9 kg in women) and overestimate FFM (md = 0.88 kg, la =  − 1.16 to 2.93 kg in girls and md = 2.9 kg, la =  − 0.19 to 6.7 kg in women) compared to DXA^31,37^.

### Assessment of arterial stiffness and blood pressure

Participants rested in a supine position for ten minutes before the measurements. Oscillometric pulse wave analysis was then performed from the right upper arm using the Arteriograph device (Arteriograph; TensioMed Ltd., Budapest, Hungary) in the supine position^33^. The device provides an automatic assessment of resting heart rate (HR), systolic (SBP), diastolic blood pressure (DBP), MAP, pulse pressure, aortic pulse wave velocity (PWVao), and augmentation index (AIx). First, the device measures actual SBP and subsequently inflates the cuff 35 mmHg above measured SBP and then measures the fluctuations in the brachial artery. The signals are passed on to a tablet computer, recorded, and analysed as pulse waves. PWVao (m/s) was calculated from the time difference between the first systolic wave (direct) and the second systolic wave (reflected) and was related to the distance from the jugulum to the pubic symphysis. AIx% was computed from the pressure difference between the first (P1) and second (P2) wave in relation to the pulse pressure by the formula AIx% = [(P2 − P1)/pulse pressure] × 100. We have previously reported a good short-term reproducibility for PWVao (intraclass correlation coefficient = 0.90, coefficient of variation = 3.7%) and moderate reproducibility for AIx% (intraclass correlation coefficient = 0.88, coefficient of variation = 29.1%) in adolescents^33^. Within the EsmiRs study sample, two repeated measurements resulted in moderate reproducibility for PWVao (intraclass correlation coefficient = 0.76, coefficient of variation = 8.4%) and AIx% (intraclass correlation coefficient = 0.68, coefficient of variation = 12.3%) when the median duration between the measurements was 2 weeks. Furthermore, Arteriograph-derived PWVao has an acceptable agreement with invasively measured PWVao in adults with a correlation coefficient between the methods 0.91 and the comparison of absolute values to provide statistically non-significant difference (mean difference − 0.05, limits of agreement 1.49 to − 1.59 m/s)^38^.

### Assessment of cardiorespiratory fitness

In the NEEDS and the EsmiRs studies CRF was assessed by a maximal ramp exercise test on an electromagnetically braked cycle ergometer (Monark 929E, Monark Exercise Ab, Sweden or Ergoselect 200, Ergoline GmbH, Germany). The NEEDS study exercise protocol included 2-min resting period sitting on an ergometer, a 2-min warm-up without resistance (0 W), and an incremental exercise period with increase of workload by 1 W/3 s (totalling 20 W/min) until voluntary exhaustion. The test was terminated when the participant was unable to keep the cadence of 50 or required to stop. The EsmiRs study exercise protocol included submaximal and maximal phases. The submaximal phase started at 20 W, and the workload increased for 20 W every four minutes until respiratory exchange ratio (RER) ⁓ 1.0 was reached. After that, participants continued directly to the maximal phase. The maximal phase started at 100 W, and the workload was increased 1 W/3 s (20 W/min) until voluntary exhaustion. The participants were asked to keep the cadence of 70–80 during the test.

In the MEndEx and the MIIA studies CRF was assessed during a maximal incremental treadmill (Telineyhtymä Oy, Kotka, Finland) or cycle ergometer test (Monark LT2, Monark Exercise AB, Vansbro, Sweden). Treadmill incline remained constant at 0.5 degrees for the entire test. Treadmill velocity was 6 or 7 km h^−1^ for the first 3-min stage of the test and was increased by 1 km h^−1^ every third min until volitional exhaustion. The cycle ergometer test was initiated at an initial power output of 50 W. After a 5 min warm-up period, the increments of 25 W were made every 2 min until exhaustion.

In all of the studies, respiratory gas exchange was assessed directly by breath-by-breath method on a metabolic cart (Vmax Encore, VIASYS Ltd., Conshohocken, USA or OxygonPro, Jaeger, Hochberg, Germany), which was calibrated before each test according to the manufacturer’s instructions.

Participants were verbally encouraged to exercise until voluntary exhaustion and the exercise tests were considered maximal if the primary and secondary objectives and subjective criteria indicated maximal effort and maximal cardiorespiratory capacity (a plateau of V̇O_2_ regardless of increasing workload, HR > 85–99% of predicted, respiratory exchange ratio ≥ 1.0/1.1, or perceived exertion in Borg 6–20 scale ≥ 18, flushing, and sweating), and the exercise physiologist supervising the exercise test considered the test maximal.

${\dot{\text{V}}\text{O}}_{{2{\text{peak}}}}$ was defined as mL × kg FFM^−1^ × min^−1^, because FFM has been considered the most appropriate normalising factor^39^. ${\dot{\text{V}}\text{O}}_{{2{\text{peak}}}}$ for mL × kg FFM^−1^ × min^−1^ was not statistically significantly associated with FFM (β =  − 0.036, 95% CI − 0.201 to 0.128, p = 0.664), indicating validity in scaling ${\dot{\text{V}}\text{O}}_{{2{\text{peak}}}}$. To allow comparison with previous studies, V̇O_2peak_ was also scaled by BM^−1^. However, ${\dot{\text{V}}\text{O}}_{{2{\text{peak}}}}$ for mL × kg BM^−1^ × min^−1^ was inversely associated with BM (β =  − 0.419, 95% CI − 0.569 to − 0.270, p < 0.001) indicating that scaling ${\dot{\text{V}}\text{O}}_{{2{\text{peak}}}}$ by BM^−1^ was not able to remove the effect of body size on ${\dot{\text{V}}\text{O}}_{{2{\text{peak}}}}$.

### Assessment of menopausal status

Menopausal status in the EsmiRs study was defined according to the Stages of Reproductive Aging Workshop + 10 guidelines using menstrual cycle and serum follicle-stimulating hormone (FSH) data as described in detail previously^35,40^. Women who self-reported not having menstrual bleeding for over 12 months and FSH level over 30 IU/L were considered postmenopausal, while women who reported still having occasional menstrual bleeding were considered peri-menopausal. All other women were considered pre-menopausal. For the present analyses, postmenopausal women with current hormone therapy were included in the pre/peri-menopausal group.

### Statistical methods

Statistical analyses were performed using the SPSS statistics software, version 28.0 (IBM Corp, Armonk, NY). Differences in the variables between adolescents, young adults, and middle-aged women were tested using the one-way ANOVA with Sidak correction for normally distributed continuous variables, the Kruskal–Wallis test for skewed continuous variables, and Chi-square test for dichotomous variables. Because PWVao and AIx% were not normally distributed, we applied logarithmic and square root transformation, respectively, before the analyses. The associations of ${\dot{\text{V}}\text{O}}_{{2{\text{peak}}}}$, BF%, and FFMI with MAP, PWVao, and AIx% and the associations of MAP with PWVao and AIx% were investigated using Linear Mixed Models. First, we investigated these associations including ${\dot{\text{V}}\text{O}}_{{2{\text{peak}}}}$, BF%, FFMI, or MAP to model as a fixed effect factor (Tables 2, 3). In Model 1, the ${\dot{\text{V}}\text{O}}_{{2{\text{peak}}}}$ data were adjusted for the exercise test modality (cycle ergometer vs. treadmill) and other data were presented unadjusted. Model 2 was adjusted for the variables included Model 1 (i.e. exercise test modality) + BF%, Model 3 was adjusted for the variables included Model 1 (i.e. exercise test modality) + MAP, and Model 4 was adjusted for the variables included Model 1 (i.e. exercise test modality) + age. Finally, we adjusted the data (Model 5) for exercise test modality (${\dot{\text{V}}\text{O}}_{{2{\text{peak}}}}$ data only), BF%, FFMI, MAP, age, and ${\dot{\text{V}}\text{O}}_{{2{\text{peak}}}}$ (mL/kg FFM/min) (body composition and MAP data only). The data on AIx% were also adjusted for resting HR^41^. Moreover, when a statistically significant associations were found in Model 5, the data were further adjusted for menopausal status.

We also investigated whether accounting for the clustered structure of data (i.e., separate cohorts in the present study) influenced the associations observed in Models 4 and 5, which were considered the main models in the present study. We used the Akaike’s Information Criterion as a measure of model adequacy, a lower value indicating a better model with optimal balance between complexity and good fit. We a priori chose the model with the lowest value of the Akaike’s Information Criterion as the final model for each outcome variable. We fitted models by allowing or ignoring possible clustering for each outcome variable. For random intercept and random slope models, we included only one variable in the random slope model (e.g., ${\dot{\text{V}}\text{O}}_{{2{\text{peak}}}}$ in the analyses on the associations of ${\dot{\text{V}}\text{O}}_{{2{\text{peak}}}}$ with outcome variables). For models 4 and 5 for all outcomes, allowing clustering did not improve model fit based on the Akaike’s Information Criterion. Furthermore, while the main interest in the basic models were to investigate the effects of covariates on the associations between independent and dependent variables, we also used the corresponding analyses for models 1 to 3 for completeness. These results have been presented in the Supplementary Material. Finally, we ran sensitivity analyses using only the data on ${\dot{\text{V}}\text{O}}_{{2{\text{peak}}}}$ assessed during cycle ergometer exercise test or body composition assessed using the InBody device and found that the results remained materially unchanged (data not shown).

**Figure 1:**
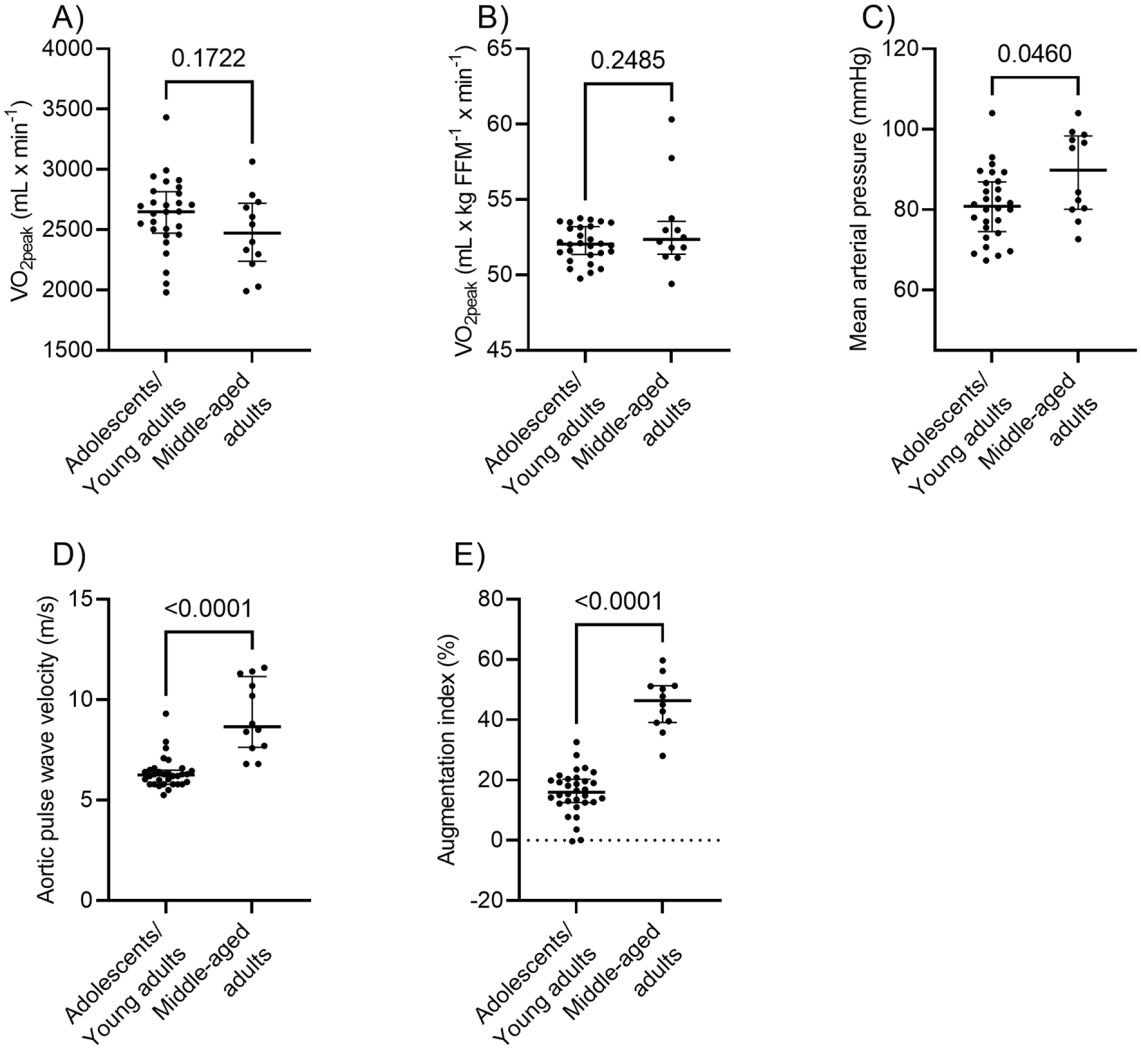
VO_2peak_, aortic pulse wave velocity, augmentation index, and mean arterial pressure among women. The women were dichotomised as adolescents/young adults and middle-aged adults.

We also investigated whether MAP, PWVao, and AIx% differ between middle-aged women with high levels of ${\dot{\text{V}}\text{O}}_{{2{\text{peak}}}}$ and adolescents and young adults with similar levels of ${\dot{\text{V}}\text{O}}_{{2{\text{peak}}}}$. Therefore, we compared MAP, PWVao, and AIx% between middle-aged women in the highest third (n = 12) of their age-specific ${\dot{\text{V}}\text{O}}_{{2{\text{peak}}}}$ scaled by FFM (49.4 to 60.3 mL × kg FFM^−1^ × min^−1^) and pooled group of adolescents and young adults with similar range of ${\dot{\text{V}}\text{O}}_{{2{\text{peak}}}}$ scaled by FFM by Mann–Whitney U-test. Because two middle aged women had higher ${\dot{\text{V}}\text{O}}_{{2{\text{peak}}}}$ scaled by FFM than most middle-aged women, we included only those adolescents and young adults with ${\dot{\text{V}}\text{O}}_{{2{\text{peak}}}}$ mL × kg FFM × min ranging from 49.8 to 53.7 (n = 32) into the analyses to allow better comparison between groups (Fig. 1, panel A,B). The α level of 0.05 was considered statistically significant.

## Results

### Characteristics

Middle-aged women were heavier than adolescents (p = 0.009) and shorter than young adults (p = 0.035, Table 1). Middle-aged women had higher BF% than adolescents (p < 0.001) and young women (p < 0.001) and they also had lower FFM than young adults (p = 0.001). Young adults also had higher FFMI than adolescents (p = 0.032) and middle-aged females (p = 0.031). Furthermore, middle-aged women had lower absolute and relative ${\dot{\text{V}}\text{O}}_{{2{\text{peak}}}}$ than adolescents and young adults (all p < 0.001). Moreover, adolescents had lower absolute ${\dot{\text{V}}\text{O}}_{{2{\text{peak}}}}$ than young adults (p = 0.013). Middle-aged women had higher SBP than young adults (p = 0.001) and higher DBP, MAP, PWVao, and AIx% than adolescents (all p ≤ 0.020) and young adults (all p ≤ 0.001).

**Table 1:** Descriptive characteristics among the study samples.

	Adolescents (n = 36)	Young adults (n = 74)	Middle-aged adults (n = 36)	p for main effect
Age (years)	17.3 (16.8 to 19.9)	21.4 (18.7 to 24.6)	55.5 (53.7 to 56.4)	< 0.001
Stature (cm)	167.6 (5.8)	169.1 (5.3)	166.4 (4.9)	0.031
Body weight (kg)	62.3 (8.6)	66.1 (8.2)	68.0 (7.5)	0.013
Body fat percentage (%)	24.9 (6.1)	22.2 (6.2)	31.2 (4.9)	< 0.001
Fat free mass (kg)	47.3 (4.0)	50.8 (5.6)	46.6 (4.5)	< 0.001
Fat free mass index (kg/m^2^)	16.8 (1.3)	17.7 (1.8)	16.8 (1.3)	0.003
V̇O_2peak_ (mL/min)	2667 (433)	2892 (363)	2154 (365)	< 0.001
V̇O_2peak_ (mL/kg FFM/min)	56.4 (7.9)	57.3 (6.8)	46.2 (6.5)	< 0.001
V̇O_2peak_ (mL/kg BM/min)	43.2 (7.2)	44.0 (5.3)	31.9 (5.4)	< 0.001
Systolic blood pressure (mmHg)	115 (109 to 121)	113 (107 to 120)	119 (115 to 129)	0.004
Diastolic blood pressure (mmHg)	67 (6.8)	64 (8.0)	73 (7.4)	< 0.001
Mean arterial pressure (mmHg)	82.9 (7.3)	80.7 (8.1)	89.6 (8.7)	< 0.001
Pulse wave velocity (m/s)	6.2 (5.8 to 6.5)	6.3 (5.9 to 6.7)	8.7 (7.6 to 10.4)	< 0.001
Augmentation index (%)	11.3 (4.2 to 14.7)	15.8 (10.3 to 20.0)	47.5 (39.1 to 51.3)	< 0.001

The data are mean (standard deviation) or median (interquartile range). The p-values are from the one-way analysis of variance or Mann–Whitney U-test.

### Associations of cardiorespiratory fitness with mean arterial pressure and arterial stiffness

${\dot{\text{V}}\text{O}}_{{2{\text{peak}}}}$/FFM was inversely associated with PWVao, and AIx% and VO_2peak_/BM was inversely associated with MAP, PWVao, and AIx% after adjustment for the testing method (Table 2, Model 1). The inverse associations of V̇O_2peak_/FFM and ${\dot{\text{V}}\text{O}}_{{2{\text{peak}}}}$/BM with PWVao and AIx% remained similar after adjustment for BF%, but the inverse association of VO_2peak_/BM with MAP was no longer statistically significant after adjustment for BF% (Table 2, Model 2). Associations of ${\dot{\text{V}}\text{O}}_{{2{\text{peak}}}}$/FFM and ${\dot{\text{V}}\text{O}}_{{2{\text{peak}}}}$/BM remained similar after further adjustment for MAP (Table 2, Model 3). VO_2peak_ scaled by FFM and BM were not associated with PWVao or AIx% after adjustment for age (Table 2, Model 4) or after full adjustments (Table 2, model 5). Further adjustment for menopausal status had no effect on these associations. V̇O_2peak_/FFM was not associated with MAP, PWVao, or AIx% in additional models.

**Table 2:** Associations of cardiorespiratory fitness with mean arterial pressure and arterial stiffness in women.

	Mean arterial pressure (mmHg)	Aortic pulse wave velocity (m/s)	Aortic augmentation index (%)
**Model 1**			
VO_2peak_ (mL/kg FFM/min)	− 0.176 (− 0.354 to 0.002)	− **0.004 (**− **0.005 to** − 0.002)***	− **0.075 (**− **0.102 to** − 0.048)***
VO_2peak_ (mL/kg BM/min)	− **0.210 (**− **0.407 to** − 0.013)*	− **0.005 (**− **0.007 to** − 0.004)***	− **0.100 (**− **0.128 to** − 0.071)***
**Model 2**			
VO_2peak_ (mL/kg FFM/min)	− 0.159 (− 0.344 to 0.025)	− **0.003 (**− **0.005 to** − 0.001)***	− **0.064 (**− **0.091 to** − 0.037)***
VO_2peak_ (mL/kg BM/min)	− 0.260 (− 0.540 to 0.021)	− **0.006 (**− **0.008 to** − 0.003)***	− **0.105 (**− **0.145 to** − 0.065)***
**Model 3**			
VO_2peak_ (mL/kg FFM/min)		− **0.003 (**− **0.004 to** − 0.001)***	− **0.060 (**− **0.084 to** − 0.036)***
VO_2peak_ (mL/kg BM/min)		− **0.004 (**− **0.006 to** − 0.003)***	− **0.080 (**− **0.106 to** − 0.053)***
**Model 4**			
VO_2peak_ (mL/kg FFM/min)	− 0.018 (− 0.215 to 0.179)	− 0.000 (− 0.002 to 0.001)	− 0.004 (− 0.017 to 0.009)
VO_2peak_ (mL/kg BM/min)	0.009 (− 0.226 to 0.244)	− 0.001 (− 0.002 to 0.001)	− 0.006 (− 0.021 to 0.010)
**Model 5**			
VO_2peak_ (mL/kg FFM/min)	− 0.021 (− 0.221 to 0.180)	− 0.000 (− 0.002 to 0.001)	− 0.006 (− 0.025 to 0.014)
VO_2peak_ (mL/kg BM/min)	− 0.025 (− 0.335 to 0.286)	− 0.000 (− 0.003 to 0.002)	− 0.005 (− 0.035 to 0.024)

The data are β coefficients and their 95% confidence intervals. Model 1: adjusted for testing method (cycle ergometer vs. treadmill), Model 2: the data were adjusted for model 1 + body fat percentage, Model 3: the data were adjusted for model 1 + mean arterial pressure, Model 4: the data were adjusted for model 1 + age; Model 5: the data were adjusted for testing method (cycle ergometer vs. treadmill), body fat percentage, fat-free mass index, mean arterial pressure, and age. Aortic pulse wave velocity was logarithmically transformed, and square root transformation was applied to Aortic augmentation index. *p < 0.05, **p < 0.01, ***p < 0.001.

Significant associations are in bold.

### Associations of body composition and mean arterial pressure with arterial stiffness

BF% was directly associated with MAP, PWVao, and AIx%, FFMI was inversely associated with PWVao, and MAP was directly associated with PWVao and AIx% (Table 3, Model 1). The inverse association between FFMI and PWVao and the direct associations of MAP with PWVao and AIx% remained statistically significant after adjustment for BF% (Table 3, Model 2). Similarly, the direct associations of BF% with PWVao and the inverse association of FFMI with PWVao attenuated but remained statistically significant after adjustment for MAP (Table 3, Model 3). Furthermore, only the inverse associations of FFMI and the direct association of MAP with PWVao remained statistically significant after adjustment for age (Table 3, Model 4) or after full adjustments (Table 3, Model 5). MAP was directly associated with AIx% in the fully adjusted model (Table 3, Model 5). In the Model 5, age was directly associated with MAP (β = 0.178, 95% CI 0.068 to 0.289, p = 0.002) and PWVao (β = 0.003, 95% CI 0.003 to 0.004, p < 0.001) and AIx% (β = 0.069, 95% CI 0.058 to 0.081, p < 0.001). Further adjustment for menopausal status had no effect on these associations.

**Table 3:** Associations of body composition and mean arterial pressure with arterial stiffness in women.

	Mean arterial pressure (mmHg)	Aortic pulse wave velocity (m/s)	Aortic augmentation index (%)
**Model 1**			
Body fat percentage (%)	0.241 (0.036 to 0.447)*	0.005 (0.003 to 0.007)***	0.087 (0.055 to 0.120)***
Fat free mass index (kg/m^2^)	− 0.195 (− 1.070 to 0.680)	− **0.010 (**− **0.019 to** − 0.002)*	− 0.070 (− 0.215 to 0.075)
Mean arterial pressure (mmHg)		0.005 (0.003 to 0.006)***	0.092 (0.069 to 0.116)***
**Model 2**			
Fat free mass index (kg/m^2^)	− 0.091 (− 0.955 to 0.774)	− **0.008 (**− **0.016 to** − 0.000)*	− 0.022 (− 0.156 to 0.122)
Mean arterial pressure (mmHg)		0.004 (0.003 to 0.006)***	0.080 (0.056 to 0.103)***
**Model 3**			
Body fat percentage (%)		0.004 (0.002 to 0.005)***	0.061 (0.032 to 0.090)***
Fat free mass index (kg/m^2^)		− **0.009 (**− **0.016 to** − 0.002)*	− 0.070 (− 0.192 to 0.052)
**Model 4**			
Body fat percentage (%)	0.030 (− 0.191 to 0.252)	0.001 (− 0.001 to 0.002)	0.010 (− 0.012 to 0.032)
Fat free mass index (kg/m^2^)	− 0.062 (− 0.881 to 0.758)	− **0.007 (**− **0.013 to** − 0.002)*	0.007 (− 0.075 to 0.089)
Mean arterial pressure (mmHg)		0.003 (0.002 to 0.004)***	0.034 (− 0.017 to 0.051)
**Model 5**			
Body fat percentage (%)	0.037 (− 0.272 to 0.197)	0.001 (− 0.001 to 0.002)	0.009 (− 0.013 to 0.032)
Fat free mass index (kg/m^2^)	0.016 (− 0.816 to 0.848)	− **0.008 (**− **0.013 to** − 0.002)**	− 0.006 (− 0.086 to 0.073)
Mean arterial pressure (mmHg)		0.003 (0.002 to 0.004)***	0.034 (0.017 to 0.051)***

The data are β coefficients and their 95% confidence intervals. Model 1: unadjusted, Model 2: the data were adjusted for model 1 + body fat percentage, Model 3: the data were adjusted for model 1 + mean arterial pressure, Model 4: the data were adjusted for model 1 + age, Model 5: the data were adjusted for body fat percentage, fat-free mass index, mean arterial pressure, age, and V̇O_2peak_ (mL/kg FFM/min). Aortic pulse wave velocity was logarithmically transformed, and square root transformation was applied to Aortic augmentation index *p < 0.05, **p < 0.01, ***p < 0.001.

Significant associations are in bold.

### Differences in arterial stiffness and mean arterial pressure in younger and older females

First, we confirmed that adolescents/young adults and middle-aged adults did not differ in absolute VO_2peak_ or VO_2peak_ scaled by FFM per (Fig. 1, panels A,B). Second, we investigated whether there were differences in PWVao, AIx%, and MAP between adolescents/young adults and middle-aged adults. Adolescents/young adults had a lower PWVao and AIx% and slightly lower MAP than middle-aged adults regardless of similar VO_2peak_ (Fig. 1, panels C–E).

## Discussion

We found that higher CRF and FFMI and lower BF% were associated with lower arterial stiffness in a sample of women aged 16–58 years. Consistent with previous studies^3,42^, we also observed that MAP was positively associated with arterial stiffness. However, the associations of CRF and BF% with arterial stiffness were largely explained by age suggesting that age-related processes leading to arterial stiffening have a more pronounced impact on arterial health than CRF and BF%. Nevertheless, the inverse association of FFMI and the positive association of MAP with arterial stiffness were independent of age and other included confounding factors indicating that maintaining sufficient muscle mass and preventing hypertension could be the key in preventing arterial stiffening and clinical cardiovascular diseases from adolescence to middle-age in women.

In line with the results of previous studies in adolescence^11,12^, young adulthood^14,15^, and middle and late adulthood^13,43,44^, we found an inverse association between CRF and arterial stiffness in women. However, age explained all reported associations between CRF, MAP, PWVao, and AIx%. While previous studies have reported an inverse association between CRF and arterial stiffness independent of age^15,45,46^, those studies have included participants from a relatively narrow age range representing participants in either adolescence, early, middle, or late adulthood. Therefore, it is possible that in our study, the sample spanning from adolescence to late adulthood, age may have a pronounced role in arterial stiffening. Some previous studies have also included both women and men^11–15^, men only^45^, or have not adjusted the data for age^44^. Therefore, it seems that due to the biological sex-differences between women and men, CRF is less important than aging itself in the process leading to arterial stiffening over the years in women. This was supported by our observations on higher arterial stiffness, as indicated by elevated PWVao and AIx%, in older women despite similar levels of CRF than younger and athletic women.

We found that a higher FFMI was associated with lower arterial stiffness independent of BF%, MAP, CRF, and age. Previous studies in older adults have also found similar associations^22–24^. However, it is not clear whether the inverse association between FFMI and arterial stiffness are due to shared genetic factors or the positive effect of resistance training on FFMI, because resistance training has been found to have weak if any effects on arterial stiffness in adults^47,48^. Furthermore, a lower hand grip strength has been associated with increased arterial stiffness in older adults^49^. It is possible that training background, for example a long-term participation in resistance training may reduce the life-course exposure to cardiometabolic risk factors potentially preventing arterial stiffening and structural changes in the arterial wall during aging^50,51^. Furthermore, a higher BF% was associated with higher arterial stiffness independent of CRF and MAP, but the association was not independent of age. While increased BF% may increase arterial stiffness through elevated blood pressure, also increased low-grade inflammation could negatively influence arterial stiffness^52,53^.

We observed that a higher MAP was directly associated with arterial stiffness independent of age. Our results agree with previous studies showing that arterial stiffness increases with increasing age and blood pressure^3,42^. The relationships between MAP and arterial stiffness may be bi-directional as higher arterial stiffness has been linked to increased risk of hypertension although other studies suggest that arterial stiffness is a consequence of high blood pressure^54^. Nevertheless, these results together suggest that the prevention of high blood pressure is essential to maintain vascular health from adolescence to late adulthood.

The strengths of our study include the sample of adolescents and young and middle-aged women, a valid and reproducible assessment of ${\dot{\text{V}}\text{O}}_{{2{\text{peak}}}}$, body composition, and arterial stiffness. However, the methods used to assess ${\dot{\text{V}}\text{O}}_{{2{\text{peak}}}}$ and body composition were not identical in all included studies, which may have a minor influence on our results. We also used the Arteriograph device that estimates only aortic PWV and AIx% and investigating other segments of arterial tree would provide additional evidence on the associations of CRF and body composition with arterial stiffness. Investigating only women removed the confounding effect of biological sex-differences. We recently found that phase of menstrual cycle, hormonal contraceptive use, or menopause status may influence arterial stiffness in adult women^35^. However, we were not able control the current data for these actors because such information was not available from all of the included original studies. Furthermore, the means of PWV and ${\dot{\text{V}}\text{O}}_{{2{\text{peak}}}}$ in our representative sample were comparable to the corresponding reference values^3,55^. Although our study included women representing adolescences, young adults, and middle adults, more studies also including women across the lifespan and women with more variable levels of arterial stiffness are warranted to investigate the associations of CRF, body composition, or blood pressure with arterial stiffness over the life course. We had limited data on physical activity, sedentary behaviour, diet quality, or traditional cardiovascular risk factors in all studies and therefore their role in the presented results remains unclear. It is possible that maintaining regular high-volume physical activity levels over the life-course attenuates or prevents arterial stiffening^56^. Finally, our study was cross-sectional and therefore causality of these associations could not be confirmed.

In conclusion, we found that a higher FFMI and a lower MAP were associated with lower arterial stiffness. We also observed an inverse association between CRF and arterial stiffness, the associations being largely explained by age. Therefore, our results suggest that maintaining muscle mass and normal blood pressure levels across the lifespan are important for vascular health in women.

## Supplementary Information

Supplementary Information.

## Data Availability

The datasets generated during and/or analysed during the current study are available from the corresponding author on reasonable request.
