# Supplementary material for: Associations of cardiorespiratory fitness, body composition, and blood pressure with arterial stiffness in adolescent, young adult, and middle-aged women
Source: Sci Rep. 2022 Dec 9;12:21378. doi: 10.1038/s41598-022-25795-x (PMC9734157; doi:10.1038/s41598-022-25795-x)
Supplement: Supplementary file 1 — Supplementary Information. [file 41598_2022_25795_MOESM1_ESM.docx]

**SUPPLEMENTARY MATERIAL**

In Models 1–3, allowing clustering did not improve the model fit, considering the data on the association between VO_2peak_/FFM and MAP. The data on the association between VO_2peak_/FFM and PWVao, FFMI and MAP, and MAP and AIx% showed the best fit with a model in which the random effect for intercept and the random slope was included. All other models showed the best fit with a model in which the random effect for intercept was included.

MAP was directly associated with PWVao and AIx% in Model 1 (Supplementary table) and the association remained statistically significant after further adjustment for BF% (Supplementary table, Model 2). FFMI was inversely associated with PWVao after adjustment for MAP (Supplementary table, Model 3) but not in other models.

| Supplementary table. Associations of cardiorespiratory fitness and body composition with mean arterial pressure and arterial stiffness in women. | | | |
| --- | --- | --- | --- |
|  | **Mean arterial pressure (mmHg)** | **Aortic pulse wave velocity (m/s)** | **Aortic augmentation index (%)** |
|  | **Model 1** |  |  |
| VO_2peak_ (mL/kg FFM/min) | -0.176 (-0.354 to 0.002) | 0.000 (-0.002 to 0.002) | -0.009 (-0.029 to 0.01) |
| VO_2peak_ (mL/kg BM/min) | -0.026 (-0.252 to 0.200) | -0.001 (-0.002 to 0.001) | -0.011 (-0.035 to 0.012) |
| Body fat percentage (%) | -0.018 (-0.241 to 0.204) | 0.000 (-0.001 to 0.002) | 0.014 (-0.009 to 0.037) |
| Fat free mass index (kg/m^2^) | 0.260 (-0.574 to 1.094) | -0.005 (-0.011 to 0.001) | 0.043 (-0.042 to 0.128) |
| Mean arterial pressure (mmHg) |  | **0.002 (0.001 to 0.004)***** | **0.032 (0.005 to 0.058)***** |
|  | **Model 2** |  |  |
| VO_2peak_ (mL/kg FFM/min) | -0.048 (-0.240 to 0.144) | 0.000 (-0.002 to 0.002) | -0.008 (-0.028 to 0.012) |
| VO_2peak_ (mL/kg BM/min) | -0.069 (-0.372 to 0.234) | -0.001 (-0.003 to 0.002) | -0.004 (-0.035 to 0.027) |
| Fat free mass index (kg/m^2^) | 0.376 (-0.508 to 1.260) | -0.005 (-0.011 to 0.001) | 0.043 (-0.041 to 0.128) |
| Mean arterial pressure (mmHg) |  | **0.003 (0.001 to 0.004)***** | **0.032 (0.005 to 0.058)*** |
|  | **Model 3** |  |  |
| VO_2peak_ (mL/kg FFM/min) |  | 0.000 (-0.002 to 0.002) | -0.010 (-0.028 to 0.009) |
| VO_2peak_ (mL/kg BM/min) |  | 0.000 (-0.003 to 0.002) | -0.011 (-0.034 to 0.011) |
| Body fat percentage (%) |  | 0.001 (-0.001 to 0.002) | 0.013 (-0.009 to 0.035) |
| Fat free mass index (kg/m^2^) |  | **-0.006 (-0.012 to -0.00006)*** | 0.025 (-0.057to 0.107) |

The data are β coefficients and their 95% confidence intervals. Model 1: adjusted for testing method (cycle ergometer vs. treadmill), Model 2: the data were adjusted for model 1 + body fat percentage, Model 3: the data were adjusted for model 1 + mean arterial pressure. Aortic pulse wave velocity was logarithmically transformed, and square root transformation was applied to Aortic augmentation index. *p<0.05, **p<0.01, ***p<0.001
